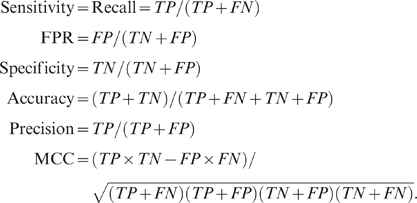# A Threading-Based Method for the Prediction of DNA-Binding Proteins with Application to the Human Genome

**DOI:** 10.1371/journal.pcbi.1000567

**Published:** 2009-11-13

**Authors:** Mu Gao, Jeffrey Skolnick

**Affiliations:** Center for the Study of Systems Biology, School of Biology, Georgia Institute of Technology, Atlanta, Georgia, United States of America; Bar Ilan University, Israel

## Abstract

Diverse mechanisms for DNA-protein recognition have been elucidated in numerous atomic complex structures from various protein families. These structural data provide an invaluable knowledge base not only for understanding DNA-protein interactions, but also for developing specialized methods that predict the DNA-binding function from protein structure. While such methods are useful, a major limitation is that they require an experimental structure of the target as input. To overcome this obstacle, we develop a threading-based method, DNA-Binding-Domain-Threader (DBD-Threader), for the prediction of DNA-binding domains and associated DNA-binding protein residues. Our method, which uses a template library composed of DNA-protein complex structures, requires only the target protein's sequence. In our approach, fold similarity and DNA-binding propensity are employed as two functional discriminating properties. In benchmark tests on 179 DNA-binding and 3,797 non-DNA-binding proteins, using templates whose sequence identity is less than 30% to the target, DBD-Threader achieves a sensitivity/precision of 56%/86%. This performance is considerably better than the standard sequence comparison method PSI-BLAST and is comparable to DBD-Hunter, which requires an experimental structure as input. Moreover, for over 70% of predicted DNA-binding domains, the backbone Root Mean Square Deviations (RMSDs) of the top-ranked structural models are within 6.5 Å of their experimental structures, with their associated DNA-binding sites identified at satisfactory accuracy. Additionally, DBD-Threader correctly assigned the SCOP superfamily for most predicted domains. To demonstrate that DBD-Threader is useful for automatic function annotation on a large-scale, DBD-Threader was applied to 18,631 protein sequences from the human genome; 1,654 proteins are predicted to have DNA-binding function. Comparison with existing Gene Ontology (GO) annotations suggests that ∼30% of our predictions are new. Finally, we present some interesting predictions in detail. In particular, it is estimated that ∼20% of classic zinc finger domains play a functional role not related to direct DNA-binding.

## Author Summary

DNA-binding proteins represent only a small fraction of proteins encoded in genomes, yet they play a critical role in a variety of biological activities. Identifying these proteins and understanding how they function are important issues. The structures of solved DNA protein complexes of different protein families provide an invaluable knowledge base not only for understanding DNA-protein interactions, but also for developing methods that predict whether or not a protein binds DNA. While such methods are useful, they require an experimental structure as input. To overcome this obstacle, we have developed a threading-based method for the prediction of DNA-binding domains and associated DNA-binding protein residues from protein sequence. The method has higher accuracy in large scale benchmarking than methods based on sequence similarity alone. Application to the human proteome identified potential targets of not only previously unknown DNA-binding proteins, but also of biologically interesting ones that are related to, yet evolved from, DNA-binding proteins.

## Introduction

The past decade has witnessed tremendous progress in genome sequencing [1]–[5]. According to the Genomes On Line Database, the complete sequenced genomes of almost 1,000 cellular organisms have been released, and about 5,000 active genome sequencing projects are on the way [6]. The unprecedented amount of genetic information has provided hundreds of thousands of protein sequences [7]. This poses a challenging problem to elucidate their functions, as experimental characterization of all newly sequenced proteins is obviously impractical. Fortunately, many of them are homologous to proteins that have been experimentally studied. Consequently, it would be highly desirable to develop computational approaches that automatically annotate a new protein sequence through its functionally characterized homologs [8]–[10]. The key component of such approaches is the ability to detect homologous relationships between un-characterized and characterized proteins. For this purpose, many sequence and structural similarity comparison methods have been developed [11]–[15]. While sequence-based methods are powerful and widely adopted for function inference [16]–[18], structure-based methods are more sensitive in detecting homologs with low or no sequence similarity [19]–[21]. However, significant sequence or structural similarity does not necessarily lead to identical function, since the functional roles of related proteins can diverge during the course of evolution [22],[23]. To address this problem, it is often necessary to examine the conservation of functionally discriminating residues when predicting enzymatic functions [17], or to evaluate the interaction energy when predicting protein-protein [24] or protein-DNA interactions [19].

DNA-binding function is a key characteristic of many proteins involved in various essential biological activities; these include DNA transcription, replication, packaging, repair and rearrangement. These DNA-binding proteins have a diversified classification according to their structures and the way they interact with DNA [25],[26]. Due to the importance of DNA-binding proteins, a few dedicated computational approaches have recently been proposed for the prediction of DNA-binding function from protein structure [19], [27]–[31]. These methods can be classified into two groups: structure template-based and template-free, depending on how (or if) they use the information from the known structures of DNA-binding proteins. Template-based methods utilize a structural comparison protocol to detect significant structural similarity between the query and a template known to bind DNA at either the domain or the structural motif level and use a statistical or electrostatic potential to assess the DNA-binding preference of the target sequence [19],[29]. The latter assessment reduces the number of false positives, which is important for the success of these methods. Template-free methods do not perform direct structural comparison, but typically follow a machine-learning framework and use features such as sequence composition and biophysical properties of surface patches [27],[28],[30],[31]. Although they can potentially detect a novel DNA-binding protein fold, template-free methods generally have lower accuracy than template-based methods, which perform well on large-scale datasets and have been applied to structural genomics targets [19].

In addition to DNA-binding function, it is also of interest to predict the amino acids that directly participate in DNA-binding. This is often straightforward for a template-based approach, as one can infer the binding residues directly from the identified template [19]. By comparison, in a template-free approach, one needs to design a new prediction protocol [32]–[36]. Recently, a new approach has been developed to predict DNA-binding residues through DNA-protein docking [37]. This approach, which takes the advantage of the non-specific DNA-binding ability of DNA-binding proteins, provides a coarse model of the DNA-protein complex in addition to the prediction of DNA-binding sites.

Although structural information is helpful for predicting DNA-binding function, it can also limit the scope of application because less than 1% of all proteins have an experimentally determined structure [38]. To overcome this limitation, we introduce a threading-based method, DBD-Threader, for the prediction of DNA-binding domains and associated functional sites. Threading-based approaches, which require only sequence as query input, have been successfully applied to the prediction of protein-protein interactions [24],[39] and protein-ligand interactions [40]. Below, we first describe the framework of our approach, and then compare its performance with three established methods, including the standard sequence alignment tool PSI-BLAST [11], the threading method PROSPECTOR [41], and the experimental structure-based DNA-binding prediction method DBD-Hunter [19]. Finally, we present the application of DBD-Threader to the human genome, for which DBD-Threader detected ∼7,000 DNA-binding domains in 59 SCOP superfamilies. We also predict that ∼20% of classic zinc finger domains play a functional role not related to direct DNA-binding.

## Results

We briefly review the general strategy of DBD-Threader (see Methods for details). Fold similarity and DNA-binding propensity are two properties employed for inferring function. Fold similarity is evaluated by a threading procedure, and the DNA-binding propensity is calculated using a statistical DNA-protein pair potential. Given the sequence of a target protein, the method first threads the sequence against a template library composed of DNA-binding protein domains whose structures have been experimentally determined in complex with DNA. Significant template hits obtained through threading, if any, are further evaluated by the DNA-protein interaction energy, calculated using the target/template alignment and the corresponding DNA structure complexed with the template protein. If a target protein has at least one significant template that satisfies both the specified Z-score and energy threshold conditions, the protein is predicted as DNA-binding and as non-DNA-binding otherwise. The threshold conditions are optimized through benchmark tests. For predicted DNA-binding proteins, DBD-Threader further assigns the SCOP superfamily to identified DNA-binding domains, provides structural models, and infers the DNA-binding protein residues according to the top-ranked template. A web-server implementation of the method is available at http://cssb.biology.gatech.edu/skolnick/webservice/DBD-Threader/.

### Functional Discriminating properties

DBD-Threader uses fold similarity evaluated by the threading Z-score, and DNA-binding propensity, evaluated by DNA-protein interaction energy, as two properties to discriminate DNA-binding proteins from non-DNA-binding proteins. The effectiveness of these two properties are demonstrated through an analysis of 179 DNA-binding proteins (DB179) and 3797 non-DNA-binding proteins (NB3797); two non-redundant datasets collected previously [19]. The sequences of these ∼4,000 proteins were used as input. For each target, we excluded from the library any template whose sequence is more than 30% identical to the target, since we are mostly interested in detecting homologs at low sequence identity.

**Figure 1:**
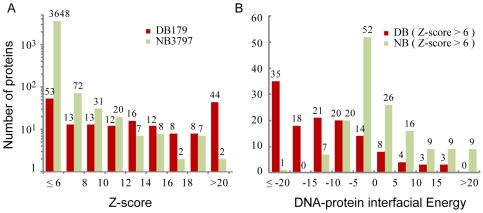
Properties selected for discriminating DNA-binding proteins from non-DNA-binding proteins. (A) Fold similarity evaluated by the threading Z-score. For each target sequence from the DB179 and NB3797 sets, the highest Z-score obtained in threading was used to create the histograms. (B) DNA-binding propensity assessed by DNA-protein interaction energy. Only significant template hits with Z-score>6 were considered. For a target with multiple hits, the one with the lowest interfacial energy was chosen to create the histogram distributions.

A significant threading Z-score for a pair of target/template proteins typically suggests a high level of structural similarity. Since two proteins with similar structures more likely share the same function than those in different structures, the threading Z-score can serve as a good indicator not only for structure similarity, but also for function similarity. As shown in Figure 1A, 70% (126) of the proteins in DB179 hit at least one template from the DNA-binding domain library with a significant Z-score>6; 25% (44) hit with a high Z-score>20. By contrast, only 3.9% (149) proteins of NB3797 hit at least one template with a Z-score>6, and only two targets from NB3797 hit a template with a high Z-score>20. These results suggest that one can utilize threading to filter out the vast majority of non-DNA-binding proteins, while keeping many homologs with DNA-binding function. However, since the numbers of proteins with a significant hit are about the same in the DNA-binding and the non-DNA-binding protein sets, about half of the predictions would be incorrect if one chooses a Z-score of 6 as the threshold to determine the DNA-binding function. One can raise the threshold to a high Z-score of 20, which would greatly improve the precision of the predictions to 96%. But, it would also reduce dramatically the sensitivity (coverage) of the predictions to only 25%. Thus, use of threading alone has a limited accuracy when applied for functional inference.

To further improve the precision without seriously compromising the sensitivity of the predictions, we introduce a DNA-protein statistical pair potential to assess DNA-binding propensity [19]. It has been shown that this term can be used to differentiate DNA-binding protein residues from non-DNA-binding residues, independent on the specific DNA substrates involved [19],[37]. If a pair of target/template proteins has similar structure, then the target protein might favorably interact with the template DNA in a similar way as the template protein. This assumption is generally valid, as shown in the distributions of the DNA-protein interaction energy of targets with at least one significant (Z-score>6) template (Figure 1B). For each target, the lowest energy is shown if more than one significant hit is identified. 94 of 126 targets from DB179 have attractive DNA-protein interaction energy values <−5, whereas only 28 of 149 targets from NB3797 have an energy value <−5. The analysis suggests that a functional relationship between remote homologs can be established at quite high precision through a combination of threading and interaction energy calculations, which is the strategy adopted by DBD-Threader.

### DNA-binding function prediction

**Figure 2:**
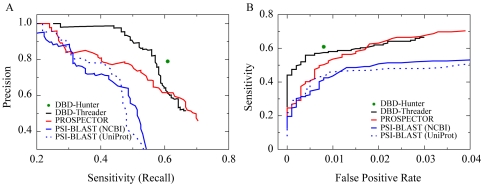
Comparison of methods for predicting DNA-binding function. Benchmark tests were performed on DB179/NB3797 sets. (A) PR (Precision *vs.* sensitivity) curves. (B) ROC (sensitivity *vs.* FPR) curves. The curves of DBD-Threader were obtained by varying the energy threshold while requiring a minimum Z-score of 6. For PROSPECTOR and PSI-BLAST, the thresholds varied to calculate the ROC and PR curves are the threading Z-score of PROSPECTOR and the E-value of PSI-BLAST, respectively. If a target hits a template with a Z-score or E-value above the specified threshold, the target is predicted as DNA-binding and non-binding otherwise. The results from DBD-Hunter were obtained with optimized parameters [19] and the same template library as used by DBD-Threader.

To benchmark the performance of our approach, DBD-Threader is compared with three methods: PSI-BLAST [11], PROSPECTOR [41], and DBD-Hunter [19]. Two sequence libraries from NCBI and from UniProt were used to derive the position specific sequence profile for PSI-BLAST, respectively. Details of the assessment procedures are given in Methods. Figure 2A shows the precision-recall (PR) and Figure 2B shows the Receiver Operator Characteristic (ROC) curves for benchmark tests on DB179 and NB3797. DBD-Threader generally performs better than PROSPECTOR and PSI-BLAST, especially at a precision higher than 0.75 and at False Positive Rate (FPR) lower than 0.01, the regime relevant to practical applications. Correspondingly, the sensitivity obtained by DBD-Threader can be higher than 0.55 within this regime. If one considers only fold similarity suggested by the threading Z-score or sequence similarity measured by the PSI-BLAST E-value, one obtains an inferior precision/FPR at the same level of sensitivity. For example, at a sensitivity value of 0.55, the precision/FPR for DBD-Threader, PROSPECTOR, and PSI-BLAST (NCBI), and PSI-BLAST (UniProt) is 0.85/0.004, 0.69/0.012, 0.24/0.085, and 0.24/0.081, respectively. Therefore, the results suggest that the quality of predictions by DBD-Threader is significantly improved when both threading Z-score and protein-DNA interaction propensity are taken into account. We also note that threading itself (PROSPECTOR) typically performs better than PSI-BLAST.

The comprehensive performance of these methods can be assessed by the Matthews Correlation Coefficient (MCC) [42]. A perfect prediction at 100% accuracy yields a MCC of one, whereas a random prediction gives a MCC of zero. The best MCCs of these four methods are provided in Table 1. The highest MCC of DBD-Threader is 0.680, corresponding to a sensitivity of 0.56 and a precision of 0.86, whereas the best MCCs of PROSPECTOR, PSI-BLAST (NCBI), and PSI-BLAST (UniProt) are 0.609, 0.540, and 0.553, both as shown in Table 1 at lower sensitivity and precision than DBD-Threader. Moreover, the best performance of DBD-Threader is only slightly lower than that (MCC 0.681) of DBD-Hunter, which requires the structure of the target as input. Note that the previous results of DBD-Hunter were obtained on a smaller template library [19]. The results reported here are based on the updated template library employed by all methods. Direct structural comparison allows DBD-Hunter to detect homology between a pair of template/target proteins with no sequence similarity, resulting in the highest sensitivity of 0.61 among all four methods at the same precision of 0.79. Nevertheless, the performance of DBD-Threader is comparable to that of DBD-Hunter in terms of its MCC. The optimal thresholds corresponding to the best performance of DBD-Threader were adopted in the application to the human genome below.

**Table 1:** Comparison of methods for predicting DNA-binding functions on DB179 and NB3797.

Method	Max. MCC		Sensitivity	FPR	Precision
**DBD-Threader**	**0.680**	0.56		0.004	0.86
**DBD-Hunter**	**0.681**	0.61		0.008	0.79
**PROSPECTOR**	**0.609**	0.53		0.009	0.74
**PSI-BLAST (NCBI)**	**0.540**	0.49		0.013	0.64
**PSI-BLAST (UniProt)**	**0.553**	0.43		0.007	0.75

The contributions by threading and by energy to the optimal performance of DBD-Threader are further dissected through an analysis of DNA-binding and non-DNA-binding proteins that share common structural folds. Here, we use the Structural Classification of Proteins (SCOP) to classify structural folds [43]. Table 2 shows the numbers of proteins (and their relevant domains) that belong to the same SCOP folds across two benchmark sets DB179/NB3797. In total, there are 109/599 proteins that contain 127/646 domains from 24 common SCOP folds. The vast majority of non-DNA-binding proteins were filtered out after the threading procedure, resulting in a 90% reduction in non-DNA-binding proteins but only a 31% reduction in DNA-binding proteins to 75/58 DB/NB proteins. After applying the optimal energy criteria, the number of DNA-binding proteins is reduced by 23% to 58, whereas the number of non-DNA-binding dramatically decreases again by 86% to 8. We note that in some sparsely populated (number of DB targets ≤4) folds, successive filtering by threading and energy left no true positive from the DB set. This is mainly due to the absence of a suitable template under the specified sequence identity cutoff of 30%. By ignoring these folds, one still obtains about 75% and 80% reduction rates on non-DNA-binding proteins through threading and energy filtering, respectively, while the majority of DNA-binding proteins are kept. Overall, the analysis shows that both threading and energy calculations significantly contribute to the ability to distinguish the DNA-binding function among proteins with similar folds.

**Table 2:** Statistics of 24 SCOP folds common to both DNA-binding and non-DNA-binding proteins in the benchmark sets.

	All		Threading		Final		
SCOP ID	N_dom_	N_pro_	N_dom_	N_pro_	N_dom_	N_pro_	SCOP Fold Description
46688	49/11	39/8	41/3	36/2	37/0	32/0	DNA/RNA-binding 3-helical bundle
52979	14/4	14/3	6/0	6/0	5/0	5/0	Restriction endonuclease-like
49379	7/26	7/23	1/0	1/0	1/0	1/0	Diphtheria toxin/TFs/cytochrome f
47768	7/1	6/1	6/0	5/0	4/0	3/0	SAM domain-like
47458	5/1	5/1	5/0	5/0	5/0	5/0	HLH-like
57943	5/44	5/44	5/15	5/15	5/3	5/3	Parallel coiled-coil
53334	4/19	4/19	4/9	4/9	1/1	1/1	SAM-dependent methyltransferases
53066	4/34	4/20	1/0	1/0	0/0	0/0	Ribonuclease H-like
54861	3/98	3/95	0/3	0/3	0/0	0/0	Ferredoxin-like
55603	6/1	3/1	6/0	3/0	1/0	1/0	Homing endonuclease-like
100938	2/1	2/1	2/0	2/0	2/0	2/0	SPOC domain-like
47953	4/10	2/6	4/8	2/4	4/4	2/2	Cyclin-like
56218	2/2	2/2	2/2	2/2	1/0	1/0	DNase I-like
57715	2/11	2/5	0/0	0/0	0/0	0/0	Glucocorticoid receptor-like
46954	2/2	2/1	2/0	2/0	0/0	0/0	Putative DNA-binding domain
81302	1/8	1/8	0/1	0/1	0/0	0/0	Nucleotidyltransferase
55944	2/13	1/13	0/4	0/4	0/2	0/2	TBP-like
55810	1/12	1/11	0/12	0/11	0/0	0/0	Nudix
50485	1/2	1/2	1/0	1/0	0/0	0/0	FMT C-term domain-like
46556	1/10	1/10	0/0	0/0	0/0	0/0	Long α-hairpin
53755	1/6	1/6	0/3	0/3	0/0	0/0	UDP-Glycosyltransferase/glycogen phosphorylase
51350	1/218	1/216	0/4	0/4	0/0	0/0	TIM α/β-barrel
50352	1/30	1/23	0/0	0/0	0/0	0/0	β-Trefoil
52539	2/82	1/80	0/0	0/0	0/0	0/0	P-loop containing nucleoside triphosphate hydrolases
**Total**	**127/646**	**109/599**	**86/64**	**75/58**	**66/10**	**58/8**	

N_dom_ and N_pro_ denote the numbers of domains and proteins from DB179/NB3797, respectively. The statistics were collected for all targets, targets detected by threading (Z-score>6), and the final positives after applying the energy filter. These three sets of numbers are indicated by All, Threading, and Final, respectively.

There are 91 non-DNA-binding proteins with at least one significant template hit (threading Z-score>6), but they are from other SCOP folds that lack any known DNA-binding protein. These non-DNA-binding proteins may contain structural fragments similar to their significant template hits or may be falsely identified by threading. By applying the energy criteria, 82 of these 91 proteins were correctly filtered out as non-DNA-binding proteins. The energy calculations, therefore, serve to reduce the number of potential false positives generated by threading.

The contribution of energy filtering can be illustrated through two examples from the NB3797. The top ranked template hits by these two targets are significant with Z-scores over 20, but these templates did not satisfy the energy criteria because of their high repulsive DNA-protein interaction energies. Both proteins are classified as non-DNA-binding. The first example is an inositol polyphosphate 5-phosphatase (PDB 1i9yA), which hits a DNA repair protein APE1 (1dewB). They are evolutionarily related and belong to the same SCOP superfamily. However, they have very different selectivity for substrate, as the inositol polyphosphate 5-phosphatase is not known to bind DNA. The second example is λ lysozyme (1am7A), which hit an endonuclease (2fldA) with a high Z-score. This seems to be a false positive by threading, since the target/template pair shares no apparent structural similarity and are not related. Nevertheless, the template did not pass energy screening.

Overall, by applying energy filtration, the number of true/false positives decreases from 131/149 (after threading) to 100/17, the numbers including results from all targets with official SCOP classification, as well as those unclassified. Thus, the filtration by energy improves the precision from 47% to 86% without dramatically compromising the sensitivity.

### Structural model and functional site prediction

**Figure 3:**
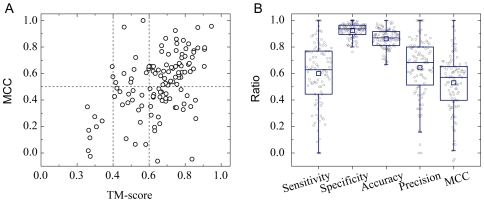
Predictions of structural models and DNA-binding sites. A total of 100 DNA-binding proteins predicted by DBD-Threader were examined individually. (A) Scatter plot of TM-score *versus* MCC. Each point represents one of 124 predicted DNA-binding domains from the 100 DNA-binding proteins. (B) Box plots overlapped with data points are shown for five performance metrics on DNA-binding protein residue predictions. Each point represents one protein including all predicted domains. The lower, middle and upper quartiles of each box are the 25th, 50th, and 75th percentile; whiskers extend to a distance of up to 1.5 times the interquartile range. Data points and means are represented by circles and squares, respectively.

In addition to function prediction, DBD-Threader also predicts structural models of DNA-binding domains from templates that provide the structural basis for function prediction. Furthermore, one may infer the functional sites directly from the template, once the functional and structural similarity between the template and the target is established. To demonstrate this point, we implemented a simple procedure in DBD-Threader that predicts DNA-binding protein residues from the top ranked template by those residues in the target aligned to DNA-binding residues in the template. In benchmark tests on DB179, this procedure was conducted on 124 domains from 100 DNA-binding proteins predicted as positives by DBD-Threader at the optimal thresholds. The value of the MCC, which measures the degree of overlap between predicted binding residues and the true binding residues observed in the native (experimental) complex structures, is used to assess the accuracy of functional site prediction. As shown in Figure 3A, DBD-Threader performs well on both structural and functional site prediction. The mean Template Modeling score (TM-score) of the top-ranked structural models of the 124 DNA-binding domains with respect to their native structures is 0.65, and 92% of these domains have a TM-score higher than 0.4, which indicates significant structural similarity [15]. Similarly, 70% of these domain models have a backbone Cα RMSD of less than 6.5 Å from their native structures. Accordingly, the mean MCC of binding site predictions is generally satisfactory, being about 0.52 for all predicted DNA-binding domains and 0.54 for domains with a TM-score higher than 0.4. As one may expect, the accuracy of binding site prediction is correlated with model quality. High quality models with a TM-score higher than 0.6 generally provide a high accuracy binding site prediction, yielding a mean MCC of 0.57, whereas low quality models with a TM-score lower than 0.4 provide inferior binding site predictions with MCCs lower than 0.4.

We further analyzed the performance according to the SCOP superfamily association of these predicted domains, as shown in Table 3. The analysis considers 84 predicted DNA-binding proteins that have an official SCOP assignment, which includes 100 domains detected by DBD-Threader and an additional 10 domains missed by DBD-Threader (see SCOP superfamily prediction below). According to their SCOP classifications, the 100 detected domains are from 31 SCOP superfamilies. The performance of DBD-Threader is generally good across various SCOP superfamilies. 24 of 31 superfamilies have a mean TM-score/MCC higher than 0.4. It appears that members of the winged helix superfamily have rather diverse DNA-binding sites. This is indicated by the mean MCC of 0.38, despite the high quality of models that are obtained (mean TM-score of 0.65).

**Table 3:** Benchmark results of SCOP superfamily predictions by DBD-Threader.

SCOP ID	True	C/U/M/I	<TM-score>	<MCC>	SCOP Superfamily Description
46689	22	16/2/2/2	0.69	0.57	Homeodomain-like
46785	14	11/2/1/0	0.65	0.38	Winged helix DBD
47113	6	6/0/0/0	0.65	0.56	Histone-fold
57959	5	2/2/0/1	0.55	0.75	Leucine zipper
47459	5	5/0/0/0	0.79	0.78	HLH DBD
47413	5	4/0/0/1	0.51	0.56	λ repressor-like DBD
56672	5	5/0/0/0	0.71	0.36	DNA/RNA polymerases
52980	5	3/2/0/0	0.53	0.25	Restriction endonuclease-like
47954	4	4/0/0/0	0.86	0.56	Cyclin-like
46894	3	2/0/0/1	0.69	0.55	C-term Domain of bipartite response regulators
56349	3	1/2/0/0	0.60	0.50	DNA breaking-rejoining enzymes
55455	2	2/0/0/0	0.67	0.79	SRF-like
47095	2	2/0/0/0	0.74	0.55	HMG-box
52141	2	1/1/0/0	0.56	0.36	Uracil-DNA glycosylase-like
100939	2	2/0/0/0	0.70	0.41	SPOC domain-like
57701	2	0/2/0/0	0.43	0.59	Zn2/Cys6 DBD
55608	2	0/1/1/0	0.61	0.49	Homing endonucleases
57667	2	2/0/0/0	0.68	0.59	C2H2 and C2HC zinc fingers
46596	1	0/0/1/0	**−**	**−**	DNA topoisomerase I, dispensable insert domain
56219	1	1/0/0/0	0.69	0.45	DNase I-like
49417	1	1/0/0/0	0.71	0.61	p53-like transcription factors
81624	1	1/0/0/0	0.78	0.75	N-term domain of MutM-like
46946	1	1/0/0/0	0.72	0.54	S13-like H2TH domain
56741	1	0/0/1/0	**−**	**−**	Eukaryotic DNA topoisomerase I, N-term DBD
48150	1	1/0/0/0	0.79	0.85	DNA-glycosylase
81585	1	0/1/0/0	0.82	0.82	DNA polymerase β-like, second domain
47802	1	0/0/0/1	0.48	0.39	DNA polymerase β, N-term domain-like
48295	1	0/0/0/1	0.27	0.17	TrpR-like
57716	1	0/0/1/0	**−**	**−**	Glucocorticoid receptor-like (DBD)
81301	1	0/0/1/0	**−**	**−**	Nucleotidyltransferase
53335	1	0/1/0/0	0.83	0.61	SAM-dependent methyltransferases
100879	1	0/1/0/0	0.80	0.79	Lesion bypass DNA polymerase, little finger domain
48334	1	0/1/0/0	0.82	0.68	DNA repair protein MutS, domain III
47823	1	0/1/0/0	0.68	0.52	λ integrase-like, N-term domain
47781	1	0/1/0/0	0.30	0.27	RuvA domain 2-like
55811	1	0/0/1/0	**−**	**−**	Nudix
53098	1	0/0/1/0	**−**	**−**	Ribonuclease H-like
**Total**	**110**	**73/20/10/7**			

The results are based on 110 DNA-binding domains from 84 predicted DNA-binding proteins that have official (True) SCOP assignments. The numbers of SCOP domain predictions are shown for four groups: Consistent (C) Un-annotated (U), Inconsistent (I), and Missed (M). Consistent predictions have the same SCOP superfamily assignments as the true assignments. If a DNA-binding domain is predicted based on a template without an official SCOP assignment, then the SCOP prediction is skipped and the domain belongs to the Un-annotated group. A target protein may contain multiple DNA-binding domains, and any domain not detected by DBD-Threader is a Missed domain. The remaining SCOP predictions, in which SCOP predictions are different from the official assignments, are considered Inconsistent. < > denotes mean. TM-scores and MCCs of DNA-binding residue predictions were calculated for all predicted domains per SCOP superfamily.

The performance measures, sensitivity, specificity, accuracy and precision, were also calculated for each of 100 proteins including all DNA-binding domains. As shown in Figure 3B, for 61% of predicted DNA-binding proteins, good functional site predictions were obtained at a MCC higher than 0.50. On average, a MCC of 0.53, a sensitivity of 0.60, a specificity of 0.93, an accuracy of 0.86 and a precision of 0.64 were obtained. The results imply that DNA binding residues were identified with satisfactory accuracy in most cases.

### SCOP superfamily prediction

The homologous relationship between the target/template pairs identified by DBD-Threader was further validated using their SCOP superfamily classifications [43]. Here, we test the idea of inferring the SCOP superfamily identity of a predicted DNA-binding domain from its templates. Among 100 predicted DNA-binding proteins, we only consider those whose SCOP assignments have been officially assigned. The consideration leads to 84 proteins composed of 110 true domain assignments, which are then compared with the predictions by DBD-Threader. The predictions can be classified into four groups, as shown in Table 3. The first group is 73 SCOP superfamily predictions that are consistent (C) with the official SCOP assignments. These correctly predicted domains are from 21 different SCOP superfamilies, including two of the most populated superfamilies, homeodomain-like and winged helix domains, with 16 and 11 correct predictions, respectively. These 27 domains consist of diverse members from 16 different SCOP families. The second group of predictions is 20 DNA-binding domains correctly identified as DNA-binding, but their SCOP superfamily classifications were un-annotated (U) because the corresponding templates have no official SCOP assignment. In 16/20 un-annotated cases, significant structural similarities between target/template pairs were found at a TM-score>0.5, implying that most of these pairs likely belong to the same superfamily. The 16 cases that are un-annotated combined with the 73 consistent SCOP predictions lead to 89 cases, or 81% of 110 domains, that may be considered correct. The third group is comprised of ten missed (M) DNA-binding domains, which are from proteins with multiple DNA-binding domains. In these cases, DNA-binding function can be successfully predicted by identifying some but not all of its DNA-binding domains. The fourth group of predictions are from the seven cases where the SCOP superfamily predictions are inconsistent (I) with the true SCOP assignment. Inspection of these predictions suggests potential functional homology in 5/7 cases. Two are presented in detail below.

**Figure 4:**
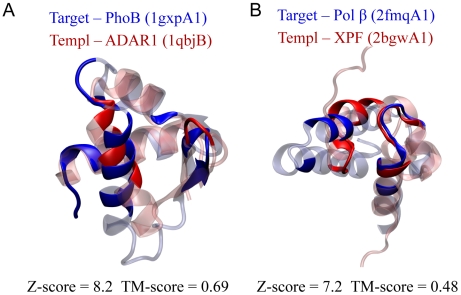
Two examples from SCOP superfamily predictions inconsistent with the official assignment suggest remote homology between the target and the corresponding template. (A and B) The structures of proteins are illustrated in cartoon representations, and colored in blue and red for the target and the template, respectively. DNA-binding and non-DNA-binding regions are shown in solid and transparent modes, respectively. For each protein, the six-character SCOP domain access code (shown in parentheses) includes a four-digit PDB code (lower case), a chain identifier (upper case), and a numeric domain identifier defined by SCOP. Molecular images were made with the program VMD [68].

In the first example, the target protein is the DNA-binding domain of PhoB, a transcription activator from *E. coli* [44]. According to SCOP, this domain belongs to the superfamily named C-terminal effector domain of the bipartite response regulators. DBD-Threader predicts that the domain belongs to the superfamily of winged helix DNA-binding domains based on its top ranked template, the Zα domain of an enzyme ADAR1 (Adenosine Deaminase Acting on RNA) from human [45]. Although ADAR1 is best known as an RNA binding protein, it is also known to bind Z-DNA with its Zα domain, as shown in multiple crystal structures of ADAR1/DNA complexes [45],[46]. In addition, the DNA-binding ability of Zα has been used to detect stable Z-DNA segments in the human genome [47], and has been linked to a new functional role of ADAR1 as a sensor of immunoreactive DNA [48]. Despite the difference in SCOP superfamily classification, the target and the template share a similar structural motif, with a high TM-score of 0.69, as shown in Figure 4A. In fact, both structures are members of the same superfamily of winged helix domains according to CATH, a hierarchical classification of protein domain structures [49]. In addition, both DNA-binding domains have similar DNA-binding sites, which include six residues from a α helix and a β hairpin (Figure 4A). The significant structural similarity and the overlap of the DNA-binding sites suggest that these two domains might have remote homology, despite the lack of sequence similarity. Thus, we have an interesting case of the PhoB domain being correctly assigned as DNA binding through the matching to an RNA binding protein that is also known to bind DNA.

In the second example, the target is the N-terminal domain from a eukaryotic DNA polymerase, Pol β [50]. The target hits a significant template from an archaeal endonuclease XPF, whose structure is composed of two heterogeneous domains [51]. As shown in Figure 4B, the target domain from Pol β was aligned to the N-terminal domain of XPF with significant structural similarity, having a TM-score of 0.48, and considerable overlap of DNA-binding residues, despite the fact that the two domains have different superfamily classifications in SCOP. The structural and functional site analysis suggests that the two domains may have a remote relationship.

### Application to the human genome

**Figure 5:**
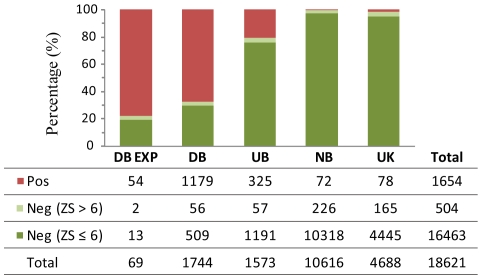
DNA-binding proteins in the human genome. DNA-binding proteins predicted by DBD-Threader are compared to the GO annotations. Pos and Neg denote positive and negative predictions by DBD-Threader, respectively.

To demonstrate that DBD-Threader is a useful tool for automatic function annotation, we applied DBD-Threader to 18,621 unique protein sequences from the human genome. The method made positive predictions for 1,654 (8.9%) proteins (see Methods for availability). Our predictions are compared to the GO annotations for the human genome [52] in Figure 5. According to the GO molecular function annotations, all human proteins can be classified into four sets: DB−1,744 proteins annotated as DNA-binding, UB−1,573 proteins not explicitly annotated as DNA-binding but annotated with a molecular function likely implicating DNA-binding, such as transcription factor activity, NB−10,616 proteins with at least one molecular function annotation and not in either DB or UB, and UK−4,688 proteins with unknown molecular function. While the vast majority of entries in DB are classified based on electronic annotations, we collected a small subset of DB, named DB EXP, in which the DNA-binding function has been verified for each member in direct experimental assay. This DB EXP set consists of 69 sequences. DBD-Threader detected at least one significant structural template for 56 of them and correctly predicted 54 as DNA-binding. Similarly, when applied to the DB set, DBD-Threader found at least one significant template for 1,235 sequences and predicted 1,179 (95%) of them as DNA-binding proteins. Notably, when applied to the UB set, DBD-Threader predicted 325 DNA-binding proteins. Among these UB positives, 256 and 51 have transcription factor activity and RNA-binding activity according to their GO annotations, respectively. These proteins likely possess DNA-binding function as well. While 89% of the positives are from either DB or UB, very few positives, 72 (0.68%), are from the NB set. This result is expected, since the chance that a protein has both DNA-binding and an unrelated molecular function is small. Despite the fact that 298 targets from NB hit a significant structural template that binds DNA with Z-score>6, 75% of them are filtered out by the interaction energy criterion. These negatives likely possess a fold similar to a DNA-binding domain, but they do not carry out the same function. Furthermore, DBD-Threader predicts 78 DNA-binding proteins among previously uncharacterized sequences. These predictions provide potentially interesting targets for further experimental validation.

A total of 6,896 DNA-binding domains from 59 SCOP superfamilies were located by DBD-Threader in the sequences of 1,654 positives. The top twenty most populated SCOP superfamilies of predicted DNA-binding domains are listed in Table 4. Notably, zinc-fingers appear in about 41% (674) of predicted DNA-binding proteins, and this particular superfamily dominates the domain predictions at 80% of the total (5,504). The second and third most common SCOP superfamilies are homeodomain-like and winged helix domains, which are found in 263 and 143 sequences, respectively. Many DNA-binding proteins, particularly zinc-fingers, contain two or more DNA-binding domains. Moreover, it is not uncommon that a sequence encodes DNA-binding domains from different SCOP superfamilies. In our annotations, we found 175 such cases.

**Table 4:** Top 20 most populated DNA-binding domains detected in the human genome.

SCOP ID	DBD-T	Pfam	N_c_	SCOP Superfamily Description
57667	5504/674	6071/688	4485/655	C2H2 and C2HC zinc fingers
46689	311/263	314/276	266/246	Homeodomain-like
46785	148/143	102/98	101/98	Winged helix DBD
57716	93/58	68/62	54/54	Glucocorticoid receptor-like DBD
47459	87/87	104/103	85/85	Helix-loop-helix DBD
47113	68/67	56/56	54/54	Histone-fold
47095	57/38	61/50	40/38	HMG-box
47413	53/50	21/21	19/19	λ repressor-like DNA-binding domains
57959	49/49	45/45	44/44	Leucine zipper domain
47954	44/26	4/2	4/2	Cyclin-like
49417	42/41	42/42	39/39	p53-like transcription factors
56672	31/28	21/21	21/21	DNA/RNA polymerases
52540	23/22	21/19	19/19	P-loop containing nucleoside triphosphate hydrolases
56219	22/19	26/26	19/19	DNase I-like
46894	22/22	0/0	0/0	C-term domain of the bipartite response regulators
117018	17/17	3/3	3/3	ATP-dependent DNA ligase DBD
56091	17/17	3/3	3/3	DNA ligase/mRNA capping enzyme, catalytic domain
50249	16/16	3/3	3/3	Nucleic acid-binding proteins
53098	12/12	10/10	10/10	Ribonuclease H-like
81296	11/11	27/22	10/10	E set domains

The numbers of domains/proteins detected by DBD-Threader and by Pfam are given for each SCOP Superfamily. The results of Pfam were obtained from the UniProt knowledge base and mapped to SCOP superfamilies (see text). N_c_ denotes the numbers of domain/proteins detected by both methods.

Our predictions are compared with Pfam predictions, which are based on Hidden Markov Models (HMMs) [53] in Table 4. The results of Pfam predictions were obtained from the UniProt knowledge base. For an objective comparison, we consider Pfam families defined for DNA-binding proteins from our template library. The Pfam definitions of these template structures were initially obtained from the PDB. These were then manually curated to ensure that the definitions correspond to the DNA-binding domains. This led to 179 Pfam families that likely include all DNA-binding proteins with known atomic complex structures, but not those with unknown structures or with only DNA-unbound structural forms. Using the SCOP definitions of the templates, we are able to assign these Pfam families to 69 SCOP superfamilies. Overall, Pfam found 7,162 significant domain matches in 1,591 proteins from the same sequence set scanned by DBD-Threader. The numbers are consistent with the 6,896/1,654 domains/proteins predicted independently by DBD-Threader.

As shown in Table 4, the results of the top three most populated SCOP superfamilies are comparable between these two methods. About 80%/85%, 4.5%/4.3%, and 2.1%/1.4% of predicted domains are zinc finger, homeodomain, and winged helix proteins by DBD-Threader/Pfam, respectively. The zinc finger proteins dominate both predictions, and over 95% of predicted zinc finger proteins were positively hit by both methods. Despite the similarity, however, about 26% of zinc finger domains detected by Pfam are predicted as negatives by DBD-Threader. One interesting question is whether these domains have evolved their function from DNA-binding to have other roles not involving DNA-binding. Although the vast majority of these proteins have not been experimentally studied, we found a few potential examples of such zinc finger domains with experimental evidence from the literature (see Case Studies below). Moreover, we noticed that DBD-Threader predictions are more diverse in terms of the number of SCOP superfamilies detected (59 *vs.* 48) and are about three times more sensitive than Pfam in assigning putative DNA binding ability to functionally uncharacterized proteins (78 positive hits *vs.* 20).

**Figure 6:**
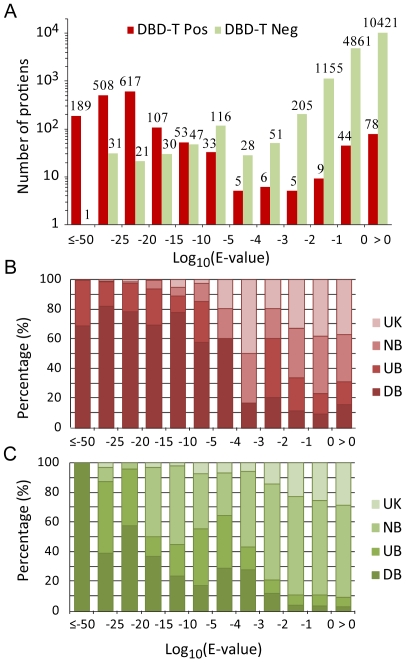
Comparison of DBD-Threader with PSI-BLAST on the human genome. (A) For each target sequence from the human genome, the lowest PSI-BLAST E-value among all target/template sequence alignments is selected to create histograms for all positive and negative DBD-Threader predictions, respectively. Breakdowns of (B) positive and (C) negative DBD-Threader predictions according to GO annotations.

Predictions of DBD-Threader are further compared with PSI-BLAST results in Figure 6. For each target, we identified the lowest PSI-BLAST E-value of all sequence alignments with all templates. In Figure 6A, the distributions of the lowest PSI-BLAST E-values are given for both positives and negatives predicted by DBD-Threader. One can immediately recognize that most positives share significant sequence similarity with a known DNA-binding domain. About 79% (1,314) of positives hit a significant template at a PSI-BLAST E-value<10^−20^. In contrast to positives, only 0.3% (53) of negatives fall into this significant E-value regime. Using the GO annotations, we found that 78% of the 1,314 positives belong to the DB set, while only 49% of the 53 predicted negatives belong to DB. On the other hand, the overwhelmingly majority of negatives (16,642) are found within the regime where the E-value is higher than 10^−3^. However, DBD-Threader managed to predict 136 positives in this regime, despite low/no sequence similarity. Analysis of their GO annotations found that 13% (18) of positives belong to DB, the ratio is over four times 2.9% (476), the rate of negatives classified as DB in the same E-value regime. The comparison suggests that DBD-Threader considerably enriches the predictions of true positives compared to PSI-BLAST.

### Case studies

DBD-Threader can make a strong prediction without apparent sequence similarity. This is illustrated through an application to the origin recognition complex subunit 6 (Orc6), which is a component of the heterohexameric origin recognition complex (ORC). The main function of ORC is to initiate DNA replication, which necessitates DNA-protein interactions [54]. It has been shown experimentally that Orc6 of *Drosophila melanogaster* binds to DNA [55]. Human Orc6 has a statistically significant sequence similarity to *Drosophila* Orc6 (PSI-BLAST E-value = 10^−24^), though the global sequence identity is relatively low at 30% over ∼240 AAs. It is not clear, however, whether human Orc6 has a similar DNA binding function [55],[56]. The sequence of human Orc6 was assessed by DBD-Threader, which predicted two DNA-binding domains in the N-terminal region (residues 1–202), based on a significant hit to the transcription factor TFIIB at a Z-score of 26 and an energy value of −9.3. By contrast, neither PSI-BLAST nor Pfam can detect a significant template from our library, which is not surprising given that there is no apparent sequence similarity between TFIIB and Orc6. Although the structure of Orc6 has not been experimentally solved, our prediction agrees with a structural model of *Drosophila* Orc6 that was recently proposed [57]. In addition, point mutations of Ser72 and Lys76, two residues located within a putative DNA-binding helix-turn-helix motif and conserved between human and *Drosophila*, abolish the DNA-binding ability of *Drosophila* Orc6 [55].

It is well-known that function inference based on sequence or structural comparison, even at a statistically significant level of similarity, can be misleading [8],[20]. By applying the energy based filter, DBD-Threader can reduce false positives generated from structural or sequence similarity comparison. This is illustrated through a second example, the barrier-to-autointegration factor-like (BAF-L) protein, whose sequence is about 40% identical to that of BAF, a known DNA-binding protein [58]. The homologous relationship was detected by PSI-BLAST (E-value<10^−46^), Pfam (E-value<10^−48^), and DBD-Threader (Z-score = 35). The GO annotations of BAF-L include DNA-binding function, probably inferred from BAL based on sequence similarity. However, using the energy filter, DBD-Threader predicts that BAF-L is not a DNA-binding protein due to its repulsive DNA-protein interaction energy. The prediction is supported by an experimental study which suggests that the functional role of BAF-L is not DNA-binding [59]. Instead, it is proposed to be a regulator of BAF through dimerization with BAF. The prediction is also supported by the fact that most residues involving DNA-binding of BAL are not conserved in BAF-L.

Particularly interesting are the classic (C2H2/C2HC type) zinc finger domains found in 41% of predicted DNA-binding proteins. The classic zinc finger domain is one of most abundant protein domains encoded in the human genome. According to the domain annotations in the UniProt knowledge base, these are 6,873 C2H2/C2HC zinc finger domain matches in 751 protein sequences of the 18,621 sequences scanned by DBD-Threader. The vast majority (92%) of these sequences contain multiple zinc finger domains. An interesting question is what functions these domains perform. If one assigns DNA-binding function according to sequence similarity detected by PSI-BLAST or Pfam, all zinc finger domains detected would be assigned as DNA-binding. Although the classic zinc finger domains originally discovered are DNA-binding domains of many transcription factors, recent studies have demonstrated that they can play a functional role through protein-protein interactions (see reviews, [60],[61]). While DNA-protein and protein-protein interactions are not necessarily mutually exclusive, it is possible that some zinc finger domains play a role involving only protein-protein interactions. About 91% of zinc finger domains annotated in UniProt were detected during threading, and 22% of these significant threading hits were assessed as negatives according to the energy calculations by DBD-Threader. Although there are inevitably false positives/negatives, we speculate that most of these negatives have acquired a functional role that does not involve DNA-binding but other biological interactions, such as protein-protein interactions.

To further examine our hypothesis, we compiled from the review in [60] a list of 18 zinc finger domains likely involved only in protein-protein interactions, as shown in Table 5. These domains, collected from six human sequences, are all experimentally well characterized. Note that we excluded domains with known DNA-binding function from these sequences. If the predictions by DBD-Threader were random, one would expect that a true negative is predicted at a success rate of 22%. Assuming that all 18 domains we collected are true negatives, we further test the null hypothesis that DBD-Threader predicts non-DNA-binding zinc finger domains at a success rate of 22% or less. Among the 18 domains, DBD-Threader predicts 4 positives and 14 negatives, which yields a significant *p*-value (7.6×10^−7^) in a one-tailed binomial test. Therefore, we rejected the null hypothesis. The result suggests that the predictions by DBD-Threader are statistically highly significant.

**Table 5:** DNA-binding assay by DBD-Threader on C2H2/C2HC zinc finger domains involved in protein-protein interactions.

Protein	ZF Domain	DBDT	Total (+/−)
FOG1	ZF1, 4§	−,+	1/1
IKZF4/Eos	ZF5,6	−,−	0/2
GLI1	ZF1	−	0/1
ZNF423/OAZ	ZF14–19	−,−,−,−,−,−	0/6
ZBT16/PLZF	ZF1,2,8,9	−,−,+,+	2/2
ZBT32/FAZF	ZF1–3	+,−,−	1/2
**Total**	**18**		**4/14**

Proteins were taken from tables 2 and 3 of reference [60]. We excluded two sequences from mouse, one (the ortholog of ZNF423/OAZ) from rat, and a long sequence (MBP-1, 2,700 AAs) not assessed by DBD-Threader. Protein EEA1 was not included because it has only one zinc finger domain and it was not detected by threading. We also discarded zinc finger domains known DNA-binding, e.g., domains from Sp1, YY1, Zac1, and BCL6. The vast majority of these DNA-binding domains, if not all, are positives. Plus and minus signs denote the positive and negative DNA-binding assessment on individual domains by DBD-Threader.

**§:** ZF4 was mistakenly labeled as ZF3 in table 2 of reference [60].

**Figure 7:**
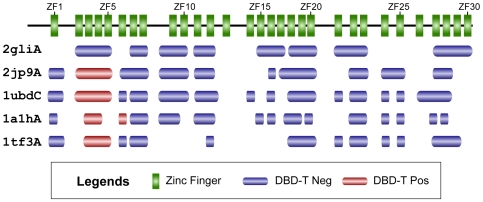
DNA-binding prediction by DBD-Threader on human OAZ. The sequence of OAZ is illustrated on top, and significant hits to five zinc-finger template proteins are shown below. These template proteins and the PDB/chain code of their structures are: GLI1 (2gliA), WT1 (2jp9A), YY1 (1ubdC), Zif268 (1a1hA), and TFIIIA (1tf3A). The green bars, blue and red squares represent zinc-fingers, negative, and positive DNA-binding predictions by DBD-Threader.

Lastly, we examine an intricate example from Table 5 in the transcription factor OAZ (Olf1/EBF-associated zinc finger protein, also known as ZNF423). This is a 1284 AA long sequence composed of 30 zinc-fingers distributed in several clusters (Figure 7). The homology of OAZ to other well-characterized zinc finger proteins, such as Zif268 and TFIIIA, were readily established by both PSI-BLAST and DBD-Threader. Significant hits with PSI-BLAST E-values<10^−20^ and threading Z-scores>15 cover virtually all zinc-finger repeats of OAZ. However, evaluation of the DNA-protein interaction energy by DBD-Threader suggests that only fingers 2 to 6 are DNA-binding, whereas other fingers do not carry out this function due to their highly repulsive energy values (typically *E*>10). The prediction is in agreement with two independent experimental studies [62],[63]. In the former study, the protein was partitioned into six clusters, and the DNA-binding activity of each was assessed with SELEX. Only the cluster containing fingers 2-5 was found to be DNA-binding [62]. The second study was performed on rat OAZ, the ortholog nearly identical (∼96%) to its human counterpart. Consistently, the DNA-binding region was mapped within the first seven fingers of OAZ [63]. In addition, both studies identified the same consensus DNA sequence recognized by these fingers. Among other zinc fingers, it was suggested that the three C-terminal zinc-fingers are essential for the interactions between OAZ and another transcription factor Olf-1/EBF, which regulates olfactory gene expression in rat [63],[64]. Another study reported that zinc-fingers 14 to 19 mediate the interaction with transcription factors Smad1 and Smad4, and that zinc-fingers 9 to 13 bind BMP (bone morphogenetic proteins) target gene promoters together with Smads [65]. The latter result that fingers 9–13 bind DNA apparently disagrees with the prediction by DBD-Threader. One possible explanation for the discrepancy is that zinc-fingers 9–13 of OAZ may adopt an atypical DNA-binding mode not present in our template library. This is supported by the observation that zinc-fingers 9–13 have unusually long (>15 AAs) linkers between them (Figure 7), whereas other structurally known DNA-binding zinc-finger proteins have shorter linkers, typically six residues, connecting their fingers. In summary, OAZ plays a central role in two distinct processes involving BMP signaling and olfactory neurogenesis, and its multi-functional role is fulfilled by different zinc fingers. While sequence or structure similarity alone cannot distinguish the functional roles of zinc-fingers, which may interact with DNA or other proteins, DBD-Threader provides a means to assess the DNA-binding preference of individual zinc-finger domains.

## Discussion

Previously, threading-based methods were proposed for predicting protein-protein and protein-ligand interactions [39],[40]. In this study, DBD-Threader extends this idea to the prediction of DNA-binding function. The method employs two key functional discriminating features: fold similarity and DNA-binding propensity. Given a target, sequence threading is used to identify a template that has a similar fold to the target. Compared with standard sequence comparison methods, such as PSI-BLAST, threading is more sensitive in detecting homology, especially when the sequence identity is lower than 30% [41]. However, since threading itself does not differentiate functional roles among sequences with a similar fold, this can give rise to a considerable number of false positives. To reduce the number of false positives, the DNA-protein interaction energy is calculated to assess whether the target preferentially interacts with DNA. In our approach, DBD-Threader uses a statistical pair potential, which has been successfully implemented in our previous application (DBD-Hunter) in predicting DNA-binding function given the native protein's structure [19]. Overall, DBD-Threader achieves better performance than approaches using only sequence homology. In benchmark tests on ∼4000 proteins, DBD-Threader is about 15% to 25% higher in sensitivity than PSI-BLAST at the same false positive rate of less than 1%, using templates that share less than 30% sequence identity with the targets. The optimal performance of DBD-Threader has a MCC of 0.68, better than the MCC of 0.61 of PROSPECTOR and 0.55 of PSI-BLAST, and is comparable to the performance of DBD-Hunter where the experimental structure of the target is required.

There exist quite a few template-free methods for predicting DNA-binding function [27],[28],[30],[31] or DNA-binding protein residues [32]–[34],[36],[37], the latter class of methods require the information that the protein is known to be DNA-binding. Most of these methods use machine-learning techniques, which provide no structural and limited biological insights. While these template-free approaches have the potential to predict the DNA-binding sites of a novel fold, their accuracy is generally lower than template-based methods [19],[37], and their performance has not been tested in large-scale benchmarks. DBD-Threader, as a template-based method, provides not only function prediction, but also structural insights into the predicted function by identifying the DNA-binding domains and associated DNA-contacting protein residues. In benchmark tests using templates with less than 30% sequence identity to the target, the backbone RMSDs of the top-ranked structural models are within 6.5 Å of their native structures for 70% of predicted DNA-binding domains. In addition, the mean sensitivity and specificity of binding site predictions is 60% and 93% among predicted DNA-binding proteins, whose DNA-binding domains have been correctly identified in terms of SCOP superfamily in most cases. The main disadvantage of a template-based approach is that it cannot predict DNA-binding function/sites for structures not present in the template library. In addition, one should generally not expect a high-level of detailed binding-site conservation between a template/target pair at low sequence identity, though the success of DBD-Threader suggests that it tends to identify functionally related template/target pairs, whose DNA-binding sites are significantly similar in most cases.

In the post-genomic era, there is a pressing need for accurate, automatic function annotation tools. DBD-Threader, implemented as a fully automated method, contributes to such a task. This is illustrated in the application of DBD-Threader to the human genome. The method predicts 1,654 DNA-binding proteins among ∼19,000 unique sequences from human. Comparing the results of DBD-Threader to their existing GO annotations, about 68% of the positives by DBD-Threader agree. Most of the remaining predictions have a GO annotation related to DNA-binding, such as transcription factor activity. Therefore, they very likely play a DNA-binding role. Moreover, DBD-Threader predicts a few protein sequences among uncharacterized sequences as DNA-binding. These can serve as candidates for further experimental examination.

The predicted DNA-binding proteins from the human genome contain 6,896 DNA-binding domains from 59 SCOP superfamilies. The vast majority of these predicted DNA-binding domains are cross-validated by other sequence annotation methods, such as Pfam annotations. The largest population of DNA-binding proteins is the zinc-finger proteins, which are about 41% of predicted DNA-binding proteins. Interestingly, 22% of detected zinc finger domains yield negative results based on the DNA-protein interaction energy assessment. Case studies of these zinc finger domains suggest that they likely perform other biological functions, such as protein-protein interactions, but not direct DNA-binding.

Function prediction from protein sequences is a challenging problem. Since proteins are evolving, they can acquire new functions and/or lose old ones. With respect to DNA-binding, a possible scenario is that it evolves to become a regulator of DNA-binding through interactions with other DNA-binding proteins, instead of directly participating in DNA-binding. While such evolution is biologically very interesting, it creates problems for approaches to function inference based on sequence similarity alone, such as those based on PSI-BLAST or HMMs. By assessing DNA-binding propensity through use of the DNA-protein interaction energy, DBD-Threader can help to discriminate DNA-binding from other functional roles, thus improving the overall quality of the predictions. Application of the method generates not only potentially interesting positives, but also negatives evolved from direct DNA-binding. Through this study, we identified 22% of zinc finger domains annotated in the human genome as such negatives with DBD-Threader.

## Methods

### Availability

All datasets listed below, the statistical potential parameters, prediction results on the human genome, and a web-server implementation of DBD-Threader are freely available at http://cssb.biology.gatech.edu/skolnick/files/.

### Data sets

#### Template library of DNA-binding domains

The PDB (April 2008 release) was queried to retrieve all protein-DNA complex structures determined by either NMR or X-ray (resolution better than 3.0 Å). The resulting 1,225 complex structures were further split into chains. SCOP annotations (version 1.73) were subsequently used to obtain domain definitions [43]. If no SCOP definition exists, the program DDOMAIN was used to partition the protein chain into domains [66]. A DNA-protein contact between a protein amino acid and a nucleotide functional group is defined if a pair of heavy atoms is within 4.5 Å, and correspondingly the protein residue is considered as DNA-binding if it contains at least one DNA-contacting heavy atom. A DNA-binding domain is selected if it has at least five DNA-binding residues. Two DNA-binding domains are considered redundant if they share more than 90% global sequence identity and more than 80% DNA-protein contact identity. The number of DNA-protein contacts and structure resolution were used to select only one representative among redundant entries, leading to a non-redundant set of 794 structures. These constitute our template library of DNA-binding domains.

#### Benchmark sets

A set of 179 DNA-binding proteins (DB179) and a set of 3,797 non-DNA-binding protein (NB3797) from a previous study [19] were used as the benchmark sets. Entries in each set are non-redundant with less than 35% sequence identity among each other. The set DB179 has also been used as the training set to derive parameters of the DNA-protein pair potential [19]. In the benchmark test on DB179, a global sequence identity cutoff of 30% was set to exclude homologous proteins from the statistical potential derivation, and the potential parameters derived individually for each target were used in the benchmarking interfacial energy calculations. The sequence identity is defined as the ratio of the number of identical residues over the length of the shorter sequence, and the sequence alignment was performed using the ALIGN0 program [67] from the FASTA2 package. For targets from NB3797, we used the parameters derived from the full set of DB179.

#### Human genome

A set of 19,293 unique protein sequences from the human genome were downloaded from the UniProt database (release version 13.3). DBD-Threader was applied to 18,621 of these sequences comprising more than 40 and less than 1,700 amino acids. Gene Ontology annotations (May 2008 release) by the European Bioinformatics Institute were used as the source for the GO annotations. Protein sequences from the human genome were classified into four sets, DB, UB, NB, and UK, according to their available GO annotations. The set DB denotes sequences annotated with a GO entry containing the key words “DNA binding”. The set DB was further culled using the evidence code IDA (Inferred from Direct Assay), resulting in a subset of DB with experimental evidence (named DB EXP). A list of molecular function entries that likely imply DNA-binding function, such as transcription factor activity, nucleotide acid binding, RNA binding, etc, was manually curated from the GO dictionary. This list was then used to build the set UB, in which each entry has at least one annotation from the list. The remaining sequences with at least one molecular function annotation, none of which involve DNA binding were assigned to the set NB. Finally, sequences with unknown molecular function belong to the set UK.

### Prediction protocol

The method DBD-Threader has three main modules: sequence threading, domain partition, and function prediction. Sequence threading was conducted using the in-house program PROSPECTOR [41]. The purpose of threading is to examine whether a target sequence encodes a structural fold similar to any structurally known DNA-binding domain. Specifically, the target sequence is threaded sequentially against two template libraries. The first library is a regular template library composed of ∼8000 protein structures, which share less than 35% global sequence identity among each other; the second library is composed of DNA-binding domains described above. The target is first threaded against the regular template library, generating statistically more robust mean and standard deviation of threading scores than threading directly on the much smaller library of DNA-binding domains. Then, the mean and standard deviation are used to calculate the Z-scores when threading templates of the DNA-binding domain library are used. Note that in the benchmark test on DB179/NB3797, we excluded all templates with more than 30% sequence identity from both threading libraries for any target. In the application to the human genome, the exclusion rule was eliminated.

For each pair of target/template proteins, a corresponding Z-score is calculated as 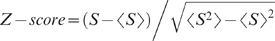
where *S* is the score associated with the best alignment between the pair, and quantity in angle brackets denotes the mean of the quantity over all entries in the regular template library. Based on our benchmark results, we consider templates with Z-scores>6 as significant hits, which are then ranked according to their Z-scores.

Since most DNA-binding proteins are composed of multiple domains, it is necessary to locate the domain(s) that directly fulfill DNA-binding function. To this end, an iterative clustering procedure was implemented to partition domains of the target sequence based on significant template hits. Clustering is required because a DNA-binding protein may contain multiple DNA-binding domains, which can hit different sets of templates. Initially, the top Z-score-ranked template is chosen as the clustering seed, and all significant templates having more than 50% overlap with respect to the seed are moved to this cluster, and excluded from subsequent clustering. After this process, if there is any template left, the highest ranked template remaining is used as a new clustering seed, and this clustering procedure is repeated until no template is left. The clustering is used to consolidate redundant templates that hit the same sequence region, and a domain can then be defined according to the alignment of a seed to the target. The threading and partition procedures are iterated for any sequence region without a significant hit that is longer than 40 amino acids, until no new domain is found. This iterative procedure can reduce missing hits to domain repeats, e.g., zinc finger clusters, because threading itself only returns the most significant alignment from each template in each round.

For function prediction, we evaluate the DNA-protein interaction energy and use it to assess DNA-binding propensity. Here, we consider only significant templates hits whose DNA-protein contacts have been obtained beforehand using the experimentally determined DNA-protein complex structures. The contacts between the target and a corresponding template DNA are inferred by replacing original template protein residues with aligned target residues. The protein-DNA interaction energy is then calculated using these contacts and a statistical pairwise potential developed previously [19]. Negative and positive energy values indicate attractive and repulsive interactions, respectively. A target is predicted to be a DNA-binding protein if at least one template yields an energy value below a specified threshold, and non-DNA-binding if no template satisfies the energy criterion. Finally, the SCOP superfamily domain assignment is inferred from the highest Z-score-ranked template that satisfies the energy criteria, and corresponding DNA-binding residues are also transferred from this template. The SCOP superfamily prediction will be skipped if the top template does not have official SCOP classification.

The optimal energy threshold values determined in benchmark tests on DB179/NB3797 are shown in Table 6. Depending on the threading Z-score of their templates, the targets fall into two regimes: Medium (20≥Z-score>6) and Easy (Z-score>20). In each regime, we select an optimal energy threshold that gives the highest MCC of predictions on DB179 and NB3797. As one expected, a more permissive energy value is obtained for the Easy targets. In the benchmarks, the ROC and PR curves of DBD-Threader were obtained by varying the energy threshold for templates in the Medium regime, and use the optimal energy threshold for templates in the Easy regime. The optimized values were adopted in the application to the human genome.

**Table 6:** Optimized Z-score and energy threshold parameters used by DBD-Threader.

Z-score Range	Energy Threshold
>20	4.6
6–20	−6.4

### Benchmark assessment

DBD-Threader was compared with three alternative approaches: DBD-Hunter [19], PROSPECTOR [41], and PSI-BLAST [11]. To ensure fair comparison, the same template library and benchmark sets DB179/NB3797 were employed. In case of DBD-Hunter, structures of targets were used as input, and the results obtained with optimized parameters are reported. When applying PROSPECTOR, the threading Z-score was used as the criterion for predictions. A target protein is classified as DNA-binding if it hits a template with a Z-score higher than a specified threshold and as a non-DNA-binding otherwise. When applying PSI-BLAST (version 2.2.17), two position specific sequence profiles were derived separately for each target using two libraries: the NCBI-NR protein sequence library (the Jul 2007 release), and the UniProt sequence library (UniRef100 version 15.5). Each profile was obtained using up to four rounds of scanning the respective libraries. We tested up to twenty rounds of iterations for profile derivation and found that four rounds gave the best performance in our benchmark tests. An inclusion E-value threshold of 0.001 and default values for other arguments were employed. For each profile generated, a final PSI-BLAST run was performed on the sequence library of the DNA-binding protein templates. If a target hits a template with an E-value higher than the specified threshold, then the target is classified as being a DNA-binding protein; otherwise, it is classified as a non-DNA-binding protein. For each target in the benchmark tests, its homologs with global sequence identity >30% were excluded from the template library of DNA-binding proteins. Note that the exclusion rule was not applied during the derivation of the PSI-BLAST profiles, and we allow all sequence hits for building the profiles.

In each prediction scenario, the numbers of true positives, false positives, true negatives and false negatives are designated as TP, FP, TN, and FN, respectively. In case of DNA-binding function prediction, a TP refers to a protein sequence correctly predicted as DNA-binding protein; in case of DNA-binding site prediction, TP refers to an amino acid correctly assigned as a DNA-binding residue. Performance measures are defined as the following: